# The external domains of the HIV-1 envelope are a mutational cold spot

**DOI:** 10.1038/ncomms9571

**Published:** 2015-10-09

**Authors:** Ron Geller, Pilar Domingo-Calap, José M. Cuevas, Paola Rossolillo, Matteo Negroni, Rafael Sanjuán

**Affiliations:** 1 Instituto Cavanilles de Biodiversidad y Biología Evolutiva, Universitat de València, C/ Catedrático José Beltrán 2, Paterna, 46980 Valencia, Spain; 2 Architecture et Réactivité de l'ARN, Université de Strasbourg, Institut de Biologie Moléculaire et Cellulaire, Université de Strasbourg-CNRS, 15 rue René Descartes, 67084 Strasbourg, France

## Abstract

In RNA viruses, mutations occur fast and have large fitness effects. While this affords remarkable adaptability, it can also endanger viral survival due to the accumulation of deleterious mutations. How RNA viruses reconcile these two opposed facets of mutation is still unknown. Here we show that, in human immunodeficiency virus (HIV-1), spontaneous mutations are not randomly located along the viral genome. We find that the viral mutation rate experiences a threefold reduction in the region encoding the most external domains of the viral envelope, which are strongly targeted by neutralizing antibodies. This contrasts with the hypermutation mechanisms deployed by other, more slowly mutating pathogens such as DNA viruses and bacteria, in response to immune pressure. We show that downregulation of the mutation rate in HIV-1 is exerted by the template RNA through changes in sequence context and secondary structure, which control the activity of apolipoprotein B mRNA-editing enzyme catalytic polypeptide-like 3 (A3)-mediated cytidine deamination and the fidelity of the viral reverse transcriptase.

## Abstract (web summary)

Mutations allow RNA virus to adapt fast but also entail fitness costs. Geller *et al*. show that, in HIV-1, mutations occur three times less often in the most external domains of the envelope, and that this is due to changes in RNA sequence context and structure, which control viral and host-encoded mutational mechanisms.

## Graphical abstract

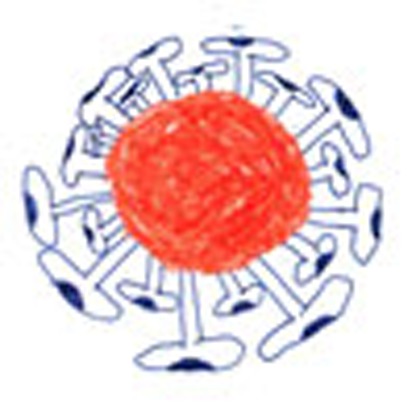

Spontaneous mutations are the ultimate source of genetic variation and are required for organisms to adapt to changing environments. Yet, mutations are more often harmful than beneficial and, therefore, their immediate effect is to reduce mean population fitness. It has been long thought that, since natural selection operates in the short term, mutation rates should tend to be minimized and approach the lower limits imposed by the efficiency of selection or the physiological costs of replication fidelity^1^,^2^. However, some organisms have evolved the ability to specifically increase their mutation rates at genome regions where selective pressure varies most rapidly, called contingency loci^3^,^4^. In bacteria, the production of mutations that improve attachment to host tissues and facilitate immune escape is promoted in surface protein-encoding genes by a sequence context rich in tandem repeats prone to polymerase slippage^5^. In contrast, bacterial mutation rates appear to have been reduced in highly expressed genes and in those undergoing strong purifying selection, although the mechanisms involved are still unknown^6^. Similarly, in vertebrates, error-prone polymerases and cytidine deaminases are responsible for somatic hypermutation of immunoglobulin genes, which allows B lymphocytes to efficiently generate high-affinity antibodies^7^. Large, slowly mutating DNA viruses can also accelerate the production of mutations in some contingency loci. For instance, in the *Bordetella* phage BPP-1, site-specific, error-prone reverse transcription is used to produce mutations in a tail fibre gene involved in host ligand recognition^8^, and similar diversity-generating retroelements have been recently discovered in bacteria^9^. Finally, vaccinia virus uses so-called genetic accordions to transiently elevate the gene copy number of the anti-host factor K3L, thereby increasing the number of mutations produced in this specific locus^10^.

RNA viruses constitute a major group of pathogens characterized by their extremely high rates of spontaneous mutation. These rates are orders of magnitude higher than those of DNA-based organisms^11^,^12^, allowing RNA viruses to evolve rapidly and conferring them a remarkable capacity to evade the immune system, become drug resistant, or colonize new hosts. However, such high mutation rates also impose a strong burden of deleterious mutations, making RNA virus populations vulnerable to extinction^13^,^14^. Although RNA viruses might benefit from targeting mutations to specific genome regions, there has been no evidence for this ability, as opposed to more complex DNA-based organisms. Viral surface envelope proteins are akin to contingency loci because they mediate attachment to host cells and are major targets of host immunity. To address whether envelope-coding RNA virus genes may experience changes in the rate of spontaneous mutation, we chose the HIV-1 envelope protein, which has been extensively characterized in terms of structure, function, antigenicity, variability and evolution. The HIV-1 envelope is formed by the external protein gp120 and the transmembrane protein gp41, and adopts a trimeric structure embedded in the virion membrane^15^,^16^,^17^. The gp120 protein is divided into five loops of extremely high genetic variability (V1–V5) interspersed with other domains that appear to be more structurally constrained and are less variable (C1–C5). Although the structure of the trimer is complex, the main targets of neutralizing antibodies tend to be located in the apical (V1–V2) and outer domains (C2–V5) of the envelope protein^18^. These domains are extensively glycosylated, allowing HIV-1 to conceal surface epitopes and thereby avoid neutralization^19^. The transmembrane gp41 protein, in contrast, is less variable, less extensively targeted by neutralizing antibodies and less glycosylated. Immune pressure is believed to be the main factor promoting sequence diversity in the viral envelope, making the V1–V5 gp120 regions the most variable region of the entire HIV-1 genome.

To test whether the HIV-1 mutation rate varies among regions of the envelope gene, we used a shuttle vector-based experimental system that allows for the accumulation of spontaneous mutations in cognate viral sequences in the absence of selection. We found that the region encoding the highly glycosylated, antibody-targeted outermost domains of the gp120 protein exhibits a threefold reduction in mutation rate compared with the rest of the envelope. Furthermore, analysis of intrapatient sequence variability data strongly suggests that this mutational cold spot also exists *in vivo*. We show that sequence context is a template-based mechanism regulating the HIV-1 mutation rate. Specifically, mutation-prone sequence motifs such as those targeted by apolipoprotein B mRNA-editing enzyme catalytic polypeptide-like 3 (A3) cytidine deaminases, are partially depleted from the gp120 external domains, therefore enabling a local reduction of the mutation rate. Furthermore, using both the shuttle vector and *in vitro* assays we found that the fidelity of the HIV-1 reverse transcriptase (RT) is dependent on RNA structure, and that differences in RNA structure in the region encoding the gp120 external domains may further contribute to lowering the viral mutation rate. These results are in sharp contrast with those obtained previously with DNA-based organisms, which tend to deploy hypermutation mechanisms at contingency loci, and unveil a paradoxical negative association between immune-driven hypervariability and the rate of spontaneous mutation in HIV-1.

## Results and Discussion

### HIV-1 mutation rate in cognate viral sequences

**Figure 1:**
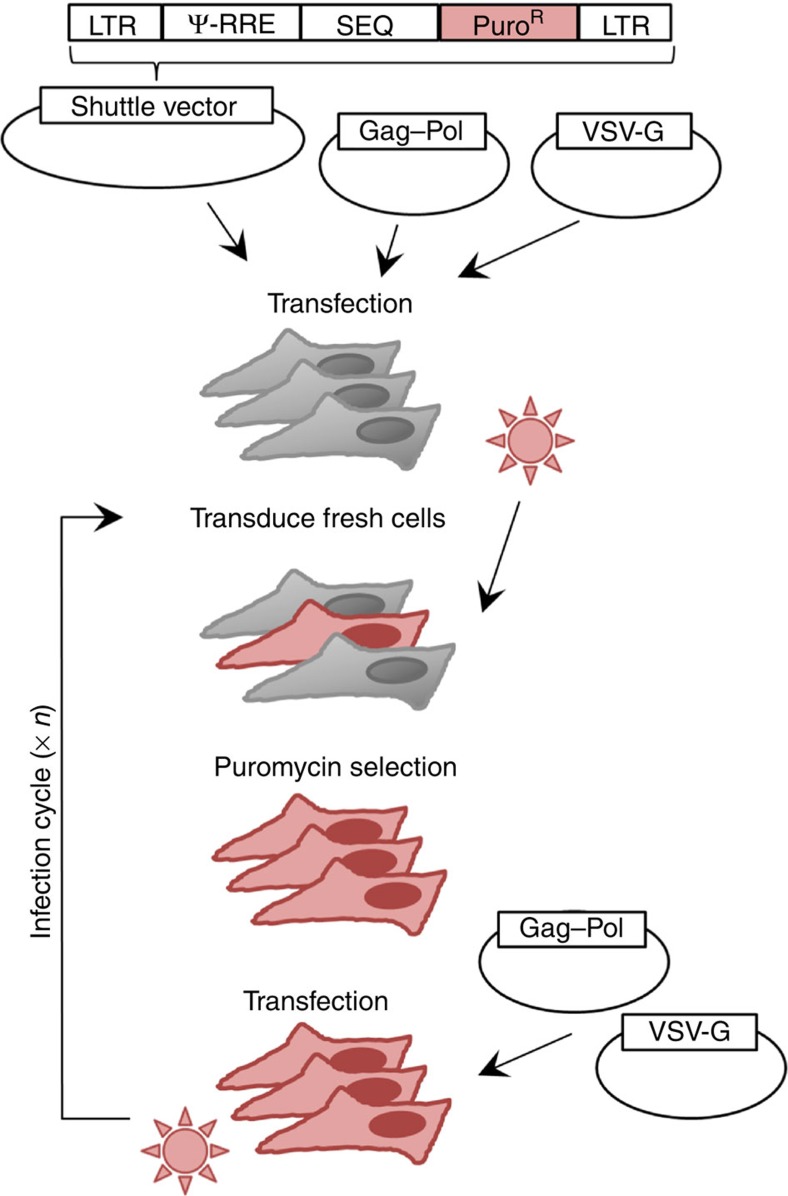
HIV-1 shuttle vector system used for scoring spontaneous mutations. A scheme of the system used for serial passaging of HIV-1 sequences in the absence of selection is shown. The shuttle vector contains the necessary elements for genomic integration (LTR) and efficient packaging (Ψ element and Rev-responsible element (RRE)), as well as the puromycin resistance gene to enable selection of cells in which integration occurs. The inserted sequence (SEQ, here *env or int–vif–vpr*) is carried forward by the vector. Translation of the Gag p17 protein starts at position 335 of our genomic RNA but, since a 2-nt insertion was introduced at position 355, the sequence rapidly falls out of frame and the SEQ insert is thus located many stop codons away from this translation initiation site (position 1,950). Translation could not start elsewhere because there is no internal ribosome entry site (IRES). The production of a protein from a spliced version of the genomic RNA is also excluded because, although the major HIV-1 splice donor site is present at position 289 of the vector, there are no splice acceptor sites in the inserted *env* sequence. Four acceptor sites are present in the *int–vif–vpr* sequence, but no protein synthesis can occur because of lack of initiating codons. The HIV-1 proteins Gag and Pol and the VSV envelope protein G are instead expressed from two helper plasmids. Initial transfection of the three plasmids is required to recover pseudotyped viruses, which are transduced into fresh cells where they undergo integration. The infection cycle can be restarted by transfecting the two helper plasmids only. After a given number of cycles, the DNA of the insert can be PCR-amplified from puromycin-selected cells, cloned, and sequenced. The inserted sequences contain no known functional *cis*-acting elements or RNA structures except for the RRE, which is required for nuclear export of viral RNA and is embedded in the *env* gene. However, this element was provided redundantly from the vector, thus minimizing selection. Recombination between vector (subtype B) and insert (subtype A) RRE copies was checked, and recombinant sequences were discarded from the analysis.

We cloned the HIV-1 subtype A envelope (*env*) gene into a shuttle vector containing the minimal *cis*-acting elements required for transcription and encapsidation. Following transfection of this construct into HEK 293T cells, pseudotyped viruses were generated by transient expression of transcomplementation plasmids encoding the HIV-1 proteins gag and pol, as well as the vesicular stomatitis virus envelope glycoprotein (VSV-G). These were then used to transduce fresh cells in which the proviral DNA became integrated, thus completing a single infection cycle. By transfecting these cells with the helper plasmids, the infection cycle was restarted, allowing for the successive accumulation of mutations (Fig. 1). The absence of selection in the cloned sequence was ensured by multiple mechanisms. First, there was no promoter driving transcription of the insert, and expression from the proviral promoter was not possible because the sequence was cloned out of frame. Second, the absence of appropriate initiation codons also excludes the possibility that spliced variants of the genomic RNA could be accidentally used for translation. Third, since HEK 293T cells lack the HIV-1 receptor and co-receptors, even a hypothetical expression of the HIV-1 envelope at the surface of the viral particles would not allow viral entry, ruling out any possible selection for functional envelopes. Finally, selection in the *cis*-acting Rev-responsive element (RRE), which is embedded in the *env* sequence and is required for nuclear export, was removed by providing a second functional copy of RRE in the genomic RNA outside *env*. To score mutations, the *env* DNA (2,598 nt) was amplified by high-fidelity PCR, cloned and sequenced after four infection cycles for each of three independent mutation accumulation lines, yielding 518 total mutations in 717,424 total bases sequenced. To verify that mutation number increased linearly with time as expected in the absence of selection, we also sequenced clones from the first infection cycle. Consistently, we found 3.9 times fewer mutations than in the fourth cycle (13 in 69,337 bases). This further rules out that mutagenesis associated with the initial transfection of the shuttle vector could have contributed significantly to the results. To also minimize the effects of random changes in mutation frequencies due to genetic drift or PCR amplification biases, within each replicate line we classified nucleotide sites as mutated or non-mutated regardless of the number of times each mutation appeared. This yielded 104 total point mutations and an estimated mutation rate of (3.6±0.7) × 10^−5^ m/n/c, which is similar to those obtained in previous studies using shuttle vector systems^12^,^20^. Of the 104 mutations observed, 93 were single-nucleotide substitutions and 11 were point insertions or deletions. Transitions were 2.2 times more frequent (64/93) than transversions (29/93), and 52% of all substitutions (48/93) were G→A changes (Table 1).

### The external envelope domains are a mutational cold spot

**Figure 2:**
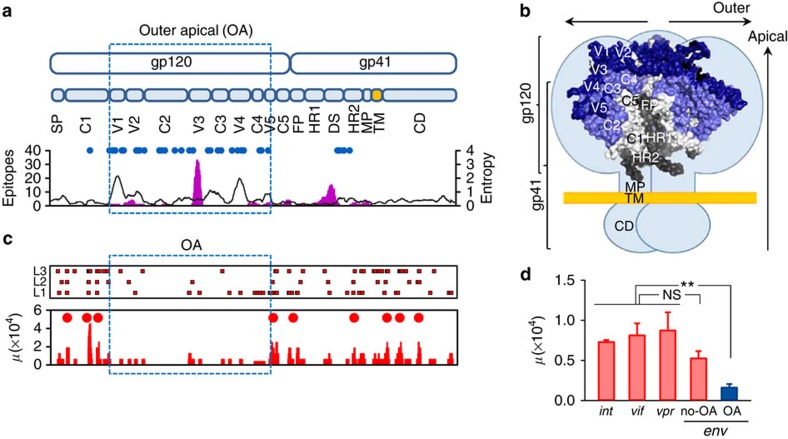
Structure of the HIV-1 envelope and location of spontaneous mutations across *env*. (**a**) Top: map of the *env* gene (SP: signal peptide; V1–V5: variable regions; C1–C5: more conserved regions between the V regions; FP: fusion peptide and FP proximal region; HR: heptad repeat; DS: disulfide loop; MP: membrane-proximal ectodomain region; TM: transmembrane domain; CD; C-terminal domain). The 1 kb region encoding the extensively glycosylated outer-apical domains of gp120 is boxed. Bottom: glycosylation sites (blue dots), number of B-cell epitopes (pink histogram), and protein sequence variability calculated as the Shannon entropy averaged over a 15-residue sliding window (black skyline). Epitopes and entropy were retrieved from the HIV Immune and Sequence Databases. (**b**) Structure of the HIV-1 envelope trimer. Each light-blue lobe represents schematically an envelope monomer embedded in the viral membrane (yellow), and superimposed is a surface representation of the crystal structure available for a portion of the trimer including most of gp120 and segments of gp41 (PDB file: 4NCO). The five variable regions are shown in dark blue, and the more conserved segments C2–C4 also belonging to the outer-apical domains are shown in slate. The three gp41 subunits are coloured in grey tones. The various regions are labelled only in one subunit of each gp120 and gp41 for clarity. The structure shown corresponds to the closed conformation found at the surface of free virions. (**c**) Top: nucleotide substitutions found in *env* for each of the lines L1–L3 after four infection cycles. Red squares indicate individual mutations. Bottom: mutation rate (*μ*) averaged over 15-base sliding window (red bars). Significant mutation clusters are indicated with red circles. (**d**) Mutation rate in the *int*, *vif*, *vpr* and *env* genes, showing the lower mutation rate in the gp120 outer-apical domains (OA, blue). **t-test: *P*<0.01; NS: not significant (*n*=3). Error bars indicate the standard error of the mean. The exact location of each mutation is provided in Supplementary Data 3 (*env*) and in Supplementary Data 4 (*int–vif–vpr*).

To address whether the spontaneous mutation rate varied along the envelope gene, we compared the observed number of mutations with the randomly expected number under the assumption of a constant mutation rate. Using a 15-nt sliding window, we found significant (Binomial test: *P*<0.01) deviation from the null expectation in 52 windows, a value twice as high as expected from type I error alone (2,598 × 0.01=25.98). Furthermore, these hits were not evenly distributed along the sequence, but were aggregated in nine clusters of consecutively significant windows. Importantly, this non-random distribution of mutations along the sequence correlated with functional and structural features of the envelope (Fig. 2). The continuous 1 kb region encoding the outer/apical (OA) glycosylated domains of gp120 (spanning from V1 to V5) showed a marked reduction in the number of mutations. Specifically, this region accumulated only 19 of the total 104 mutations, whereas the rest of *env* (1.5 kb encoding the signal peptide, the gp120 C1 and C5 regions, and gp41) showed 85 mutations (Fisher test: *P*<0.001). On the basis of these data, the calculated mutation rate of the region encoding the gp120 OA domains was (1.6±0.5) × 10^−5^, which represents a 3.1-fold reduction compared with the rest of *env*. This drop was driven by nucleotide substitutions, which occurred at 3.6-fold lower rate in the region encoding the gp120 OA domains (15 in 1.0 kb) than in the rest of *env* (78 in 1.5 kb; Fisher test: *P*<0.001; Fig. 2c), whereas point insertions/deletions appeared to be more homogeneously distributed (four in the OA region versus seven in the rest of *env*; Fisher test: *P*>0.5). This mutational cold spot was unexpected, because the gp120 OA domains contain the most variable regions of the HIV-1 genome. Therefore, counterintuitively, immune-driven HIV-1 sequence hypervariability was associated with a reduction in the rate of spontaneous mutation. For comparison, we performed similar experiments with a 1,566 nt fragment encompassing the integrase-coding region followed by the *vif* and *vpr* genes (*int–vif–vpr*), which does not contain hypervariable regions. We found 102 unique mutations after six infections cycles in 227,070 bases sequenced, including 95 single-nucleotide substitutions and 7 point insertions/deletions (Table 1). The estimated mutation rate for this region was (7.5±0.5) × 10^−5^ m/n/c, a value significantly higher than for the gp120 OA domains (*t*-test: *n*=6, *P*=0.001) and not significantly different from that of the rest of *env* (*t*-test: *n*=6, *P*=0.073; Fig. 2d). To address whether the mutation rate varied along the *int–vif–vpr* fragment, we compared the observed number of mutations against the randomly expected number, using a 15-nt sliding window as above (Supplementary Fig. 1). We found 12 windows enriched in mutations (Binomial test, *P*<0.01), a value close to the 0.01 × 1566=15.7 expected from type I error, and significantly lower than in *env* after accounting for differences in sequence length and the total number of mutations sampled (Fisher test: *P*<0.001). Therefore, unlike in *env*, there was no evidence for mutational cold spots in the *int–vif–vpr* region.

### HIV-1 mutations are dependent on sequence context

Analysis of the type of mutations produced in the shuttle vector and their sequence context can provide insight into the mechanisms underlying mutation rate variation. G→A substitutions, which were the most frequent type of change, may be in principle produced by the HIV-1 RT, which has been previously shown to exhibit a bias towards this specific substitution in some studies^21^, but not in others^22^,^23^. Importantly, though, G→A substitutions occurred 77% of the time (77/100 pooling *env* and *int–vif–vpr*) at GG or GA dinucleotides, which are the canonical sequence targets of host A3G and A3D/F/H cytidine deaminases, respectively^24^, whereas the HIV-1 RT shows no such dinucleotide preferences^21^. Previous work has demonstrated that various A3 forms are expressed at low, yet detectable levels in HEK 293T cells, and that this could lead to G→A substitutions in the absence of the virus-encoded A3 inhibitor Vif, albeit the expression level is probably not sufficient to trigger hypermutation^25^,^26^. We therefore conclude that a fraction of the observed G→A substitutions was probably produced by A3 activity. In addition to contributing a subset of G→A substitutions, the HIV-1 RT should have produced the vast majority of all other mutations types. Other possible sources of mutation include the host RNA polymerase II, which produces five to ten times fewer mutations than HIV-1 RT (refs 27, 28), as well as spontaneous nucleic acid damage. To explore the effect of sequence context beyond GG and GA motifs, we tested for associations between mutations and all possible di-, tri- and tetra-nucleotides. This revealed 16 sequence motifs for which the mutation rate was significantly increased over the background level (Table 2). In 12 of 16 such motifs, mutation enrichment was driven by G→A substitutions according to known A3 sequence context preferences^29^. Of the other four motifs, three contained the TG dinucleotide, which is also consistent with A3 edition provided that G is followed by G or A, but may also be a RT hotspot. Finally, the TTCT motif was also enriched in mutations, which were always C→T substitutions. These substitutions may have been produced by A3 acting directly on viral RNA^30^ or on the coding strand of the viral DNA^31^, or alternatively by the HIV-1 RT. Of the 16 motifs detected in *env*, 12 also showed higher-than-average propensity to mutation in the *int–vif–vpr* fragment (all except TTCT, which was absent from the fragment, GTGA, TTG and TTGG; Fisher test: *P*<0.017). Overall, these results demonstrate a dependence of the HIV-1 mutation rate on sequence context, which is mainly driven by canonical A3 preferences.

### A3 targets are partially depleted in the gp120 OA domains

**Figure 3:**
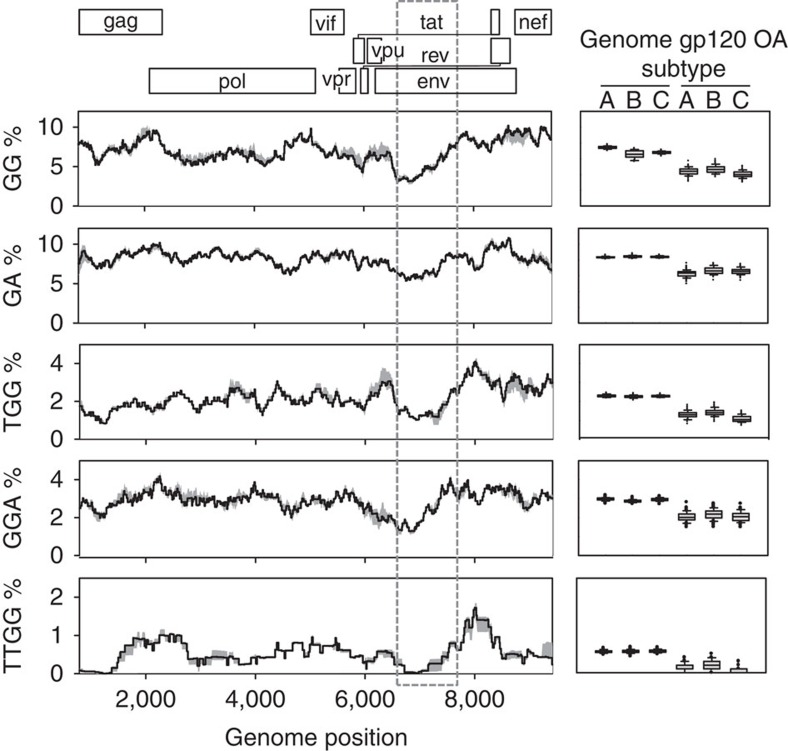
Mutation-prone A3 targets showing significant depletion in the gp120 OA domains. A map of the HIV-1 genome is shown on top, with each reading frame shown at a different level. The skyline plots represent the percent abundance of each motif averaged over a 0.5 kb sliding window for 100 sequences of each subtype A, B and C. The grey shaded area around the black line represents the range of variation among subtypes. The dashed box shows the 1 kb region encoding the gp120 OA domains. Genome positions correspond to the HXB2 sequence. To the right of each skyline plot is shown a boxplot of the motif abundance in the gp120 OA domains versus the rest of the genome, based on the 100 individual values from each subtype. The lines in the box indicate the first, second (median) and third quartile. Whiskers above and below the box indicate percentiles 10 and 90, and outlying points are individually plotted.

Since A3 probably was a significant source of mutations in our system, changes in the A3-driven mutation rate may contribute to explaining the gp120 mutational cold spot. We found that the 1 kb region encoding the gp120 OA domains accumulated only five of the 38 total *env* A3-like mutations, defined as G→A substitutions at GG or GA dinucleotides, which represents a 4.6-fold reduction compared with the rest of *env* (Fisher test: *P*<0.001; Supplementary Fig. 2). Of the 368 possible A3 dinucleotide targets in *env*, 23 were mutated in one of the replicate lines, but three were mutated in two lines and three were mutated in the three lines. The number of targets showing two or more mutations was significantly higher than expected assuming a constant per-target mutation probability (six observed versus 1.83 expected from a Poisson distribution: *P*=0.011), and targets with three mutations were also significantly overrepresented (three observed versus 0.06 expected; Poisson distribution: *P*<0.001). Interestingly, these multiply mutated sites were located in two small clusters flanking the gp120 OA domains (a 200-base region mapping to the gp120 C1 domain, and a 30-base region located in the C5 domain) suggesting that these regions were particularly prone to A3 editing. However, A3-like mutations were still 2.5 times less abundant in the 1 kb region encoding the gp120 OA domains than in the rest of *env* after removal of these recurring mutations. We found that GG dinucleotides were depleted by twofold in the gp120 OA region, thus reducing the opportunities for A3-mediated mutation, whereas GA dinucleotides showed a more modest 1.3-fold reduction in frequency. This depletion provides an additional explanation for the lower number of A3 mutations found in the gp120 OA domains. To test whether this change in sequence context was unique to our sequence, we also analysed 100 publicly available full-length sequences from each subtype A, B and C (Fig. 3). We found that the GG motif was significantly less frequent in the gp120 OA domains (4.39±0.21% across subtypes) than in the rest of *env* (7.97±0.06%, paired *t*-test: *n*=3, *P*=0.005), and that these domains consistently appeared as the region with the lowest GG abundance in the entire HIV-1 genome. The genome-wide frequency of the GA dinucleotide also floored at the gp120 OA domains, although the drop was less pronounced than for GGs. Other mutation-prone A3 target motifs also showed diminished abundance in these domains, including TGG (1.26±0.10% versus 2.27±0.01% in the rest of the genome, paired *t*-test: *n*=3, *P*=0.011), GGA (2.08±0.04% versus 2.93±0.03%, paired *t*-test: *n*=3, *P*=0.007), and TTGG (0.14±0.05 versus 0.56±0.01%, paired *t*-test: *n*=3, *P*=0.013). For triplets such as the tryptophan codon TGG, this drop cannot be explained by differences in amino acid usage because out-of-frame TGG triplets were also significantly reduced (1.42±0.08%, paired *t*-test: *n*=3, *P*=0.010). Motif abundance was indeed accounted for by base composition, since the values expected from base composition alone were similar or even lower (3.55%, 7.39%, 0.87%, 1.41% and 0.21% for GG, GA, TGG, GGA and TTGG, respectively) than observed values. This effect was mainly driven by G content, which dropped from 24 to 19% in the gp120 OA domains. Overall, these results show that sequence context provides a template-based mechanism for regulating the A3-driven HIV-1 mutation rate in the most external domains of the viral envelope.

### RNA structure determines the fidelity of the HIV-1 RT

**Figure 4:**
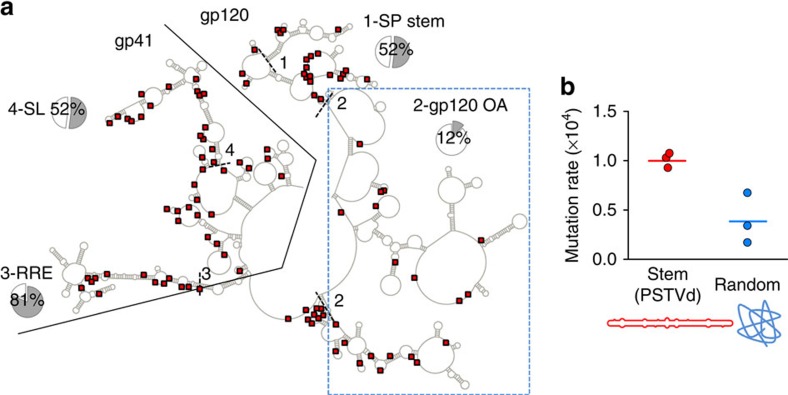
Effect of RNA structure on the HIV-1 mutation rate. (**a**) Mapping of the shuttle vector spontaneous mutations in the *env* RNA structure model. Nucleotide substitutions are represented with red squares. Regions encoding gp120 and gp41 are separated with a black line. Short dashed lines delimit the following regions in the structure: SP stem (1), region encoding the gp120 OA domains (2), Rev-responsive element (RRE, 3), and a multi stem-loop structure identified in gp41 (SL, 4). For each, the pie chart indicates the fraction of sites forming base pairs, considering only sites for which the pairing status has >90% chance of being conserved across HIV-1 subtypes. (**b**) *In vitro* mutation rate of the HIV-1 RT using a stem-like template RNA obtained from PSTVd (red) versus a randomized sequence (blue). Each dot represents an individual replicate and the horizontal bars the mean rate. A list of mutations and their location is provided in Supplementary Data 5 (PSTVd) and Supplementary Data 6 (randomized).

More than half of nucleotide substitutions detected in the shuttle vector did not show an A3-like G→A mutational signature, therefore suggesting variations in RT fidelity along the sequence. Of the 55 non-A3 nucleotide substitutions, only 10 were located in the 1 kb region encoding the gp120 OA domains, thus showing a 3.1-fold mutation rate reduction in this region compared with the rest of *env* (Fisher test: *P*=0.001; Supplementary Fig. 2). Previous work has shown that RNA secondary structure determines the propensity of RTs to slippage as well as the rate of recombination in retroviruses^32^,^33^,^34^. To investigate the possible role played by RNA structure in determining the HIV-1 mutation rate, we first mapped our shuttle vector mutations to the RNA structure published for subtype B NL4-3 sequence, which was obtained using selective 2′-hydroxyl acylation analysed by primer extension (SHAPE)^35^. In this structural model, 98.9% of the *env* sites form intragenic base pairs or are unpaired, thus suggesting that isolation of the *env* RNA transcript from the rest of the genome in our system should not have significantly altered its secondary structure. Since our sequence and NL4-3 belong to different subtypes, we restricted our analysis to the 831 sites for which the paired/unpaired status had >90% probability of being evolutionarily conserved across HIV-1 subtypes within the M group, as determined previously^35^. The gp41-coding region shows higher level of base pairing (56%) than gp120 (19%; Fisher test: *P*<0.001) or the 1 kb region encoding the gp120 OA domains, which shows very little structure (12% of sites paired; Fig. 4a). To more directly test for the effect of RNA structure on RT fidelity and remove possible confounders stemming from A3 activity or HIV-specific sequence context variations, we performed *in vitro* reactions in which the HIV-1 RT was used to reverse transcribe a 361-base RNA from the potato spindle tuber viroid (PSTVd). We chose this RNA because it exhibits a marked, stem-like, secondary structure which has been extensively characterized by various methods including SHAPE^36^. Reverse transcription products were used for high-fidelity PCR, molecular cloning and sequencing. In three replicate assays, we found 20 unique mutations (7, 7 and 6) in 199,221 total bases sequenced, giving an *in vitro* error rate of (1.00±0.04) × 10^−4^ for the highly structured PSTVd RNA. As a control, we performed the same experiments using a randomly shuffled PSTVd sequence to disrupt RNA structure. We found only 7 mutations in 178,285 total bases in the shuffled RNA, giving an error rate of (0.39±0.15) × 10^−4^, which represents a 2.6-fold reduction compared with the stem-like sequence (*t*-test: *P*=0.016; Fig. 4b). Although the number of mutations was low to confidently infer the mutational spectrum, we observed 6 transitions (including a single G→A substitution), 10 transversions and 4 point insertions in the PSTVd RNA whereas, in the randomized sequence, 6 of 7 mutations were transitions, suggesting an effect of RNA structure on the type of mutations produced. Overall, these *in vitro* assays reinforce the conclusion that RNA structure is a template-based mechanism regulating HIV-1 RT fidelity, and that the lower level of base pairing shown by the gp120 OA region probably contributes to lowering the viral mutation rate in this region relative to the rest of the envelope gene.

### The gp120 OA domains are a mutational cold spot *in vivo*

We next sought to explore whether our findings might accurately reflect the HIV-1 mutational process *in vivo* by analysing sequences from patients. For this, we used the lethal mutation method, which is based on the principle that the frequency of lethal mutations in a population depends solely on the rate of spontaneous mutation^37^. This implies that by analysing likely lethal mutations such as premature stop codons, it is possible to approximately infer the mutation rate from intrapatient sequence variability data^38^,^39^. We used two large, publicly available data sets of HIV-1 envelope sequences, both obtained from plasma samples by high-fidelity limiting-dilution PCR^40^,^41^. These two data sets yielded very similar stop codon mutation frequencies after dividing by the number of possible stop mutations, or non-sense mutation targets (NSMTs), 4.7 × 10^−5^ and 4.8 × 10^−5^ (Table 3). However, as in the shuttle vector, these mutations were not evenly distributed throughout the *env* sequence. The 1 kb region encoding the gp120 OA domains harboured only 21% (17/82) of the total observed stops despite encompassing 40% of the *env* sequence (Fisher's test: *P*=0.016). To further explore this, we carried out a similar analysis using viral DNA instead of plasma-derived sequences. We purified total DNA from PBMC pellets obtained from 10 untreated, chronically infected patients, amplified 50 individual *env* clones per patient by high-fidelity limiting-dilution PCR, and subjected them to massive parallel sequencing using the Illumina technology. Consistent with the fact that proviral DNA is an archive of non-functional viruses^42^ we obtained a frequency of stop codons nearly two orders of magnitude higher than in plasma, thus allowing us to increase our statistical power by sampling >1,000 stop codon mutations (Table 3). Again, we found that in 10 of 10 patients the frequency of stop codon mutations was significantly reduced in the gp120 OA domains compared with the rest of *env* (Fisher test: *P*<0.001 in each patient). Pooling the 10 patients, the stop codon frequency per NSMT was reduced by 4.6-fold, from 1.1 × 10^−2^ to 2.4 × 10^−3^. Therefore, analysis of viral sequences from patients confirms the existence of a mutational cold spot in the gp120 OA domains *in vivo*.

### Evolutionary interpretation

Our results contrast with previous knowledge from DNA organisms, in which contingency loci involved in host–pathogen interactions have been shown to display strong elevations of the mutation rate^5^,^7^. Similarly, rapidly changing selective pressures associated with novel environments, stress factors, immune pressure or phage infections have been shown to favour mutator strains in bacteria^43^,^44^,^45^,^46^. In slowly mutating pathogens, hypermutation mechanisms targeting contingency loci should be advantageous because they critically reduce the waiting time for the appearance of escape mutations. In contrast, in the rapidly mutating HIV-1, mutational supply should not be a major factor limiting adaptation since molecular evolutionary^47^ and experimental^48^,^49^,^50^ studies suggest that the average mutation rates of HIV-1 and other RNA viruses are close to the optimal value providing maximal adaptability. As discussed above, the HIV-1 envelope is subject to strong immune pressure, and A3 is one possible source of escape mutations^51^,^52^,^53^. However, the accumulation of A3-driven mutations will tend to deplete GG and GA targets, thus leaving fewer such targets available for subsequent mutation^53^. Therefore, over time, A3 activity combined with positive selection of escape mutants in the immune-targeted gp120 OA domains may lead to a reduction of the rate of spontaneous mutation in this specific region. A similar argument applies to RNA secondary structure, which may be disrupted by protein-level positive selection^54^, consequently changing RT fidelity. We thus suggest that mutation pressure in combination with positive selection may be at the origin of the gp120 OA mutational cold spot. Furthermore, reducing the rate of spontaneous mutation in this region may afford long-term benefits to the virus. Despite being obviously advantageous in the host where they were selected, several lines of evidence suggest that many antibody escape mutations can be costly in subsequent hosts, thus inflating the genetic load of the viral population over the long term. For instance, it has been shown that swapping the hypervariable V1–V2 domains between different primary isolates of the same subtype severely impairs envelope function and stability in more than half of the cases, revealing an interference of genetic variability with *env* function^55^. Similarly, a recent study reported that ∼50% of mutations affecting *N*-glycosylation had severely adverse effects on viral infectivity by altering protein structure and function^56^. Although the envelope glycan shield is evolvable, the amount of glycosylation stays remarkably constant among HIV-1 isolates, thus suggesting that maintenance of this shield is essential for virus survival^57^. Therefore, the fitness costs of immune-driven variability in the most external domains of the gp120 protein may be substantial, possibly favoring the long-term maintenance of sequence contexts and RNA secondary structures that down-regulate the HIV-1 mutation rate in these regions.

## Methods

### DNA constructs

Plasmids pSDY-dCK, pGag-Pol (originally named pCMVΔ8.91), and pVSV-G (originally named pHCMV-G) were obtained in previous work^33^,^34^. pSDY-dCK encodes the *cis*-acting elements required for RNA packaging and processing (Ψ element and RRE), followed by the deoxycytidine kinase gene (dCK) driven by the EF1 promoter and a puromycin resistance gene driven by the GK promoter, all of which are flanked by HIV-1 LTR sequences to allow for genomic integration. To generate the shuttle vectors encoding HIV-1 cognate sequences, PCR was performed to amplify a 2,628-bp sequence including the HIV-1A envelope gene (HXB2 positions 6,220–8,817) and a 1,566-bp sequence encompassing the integrase, *vif*, and *vpr* genes (HXB2 positions 4,230–5,796), using Phusion high-fidelity DNA polymerase. PCR primers included MluI and XhoI restriction sites, which were used to clone the PCR products into the pSDY-dCK vector using the corresponding sites in the vector, removing the EF1 promoter and dCK gene in the process (primers Env_F_Mlu/Env_R_Xho and IVV_F_Mlu/IVV_R_Xho for env and *int–vif–vpr* fragments, respectively; see Supplementary Table 1 for primer sequences). Successful insertion of the PCR products was verified by sequencing.

### Serial passaging and sequencing of the HIV-1 shuttle vector

To generate pseudotyped viruses harbouring the *env* or *int–vif–vpr* sequences, 5 × 10^6^ HEK 293T cells (ATCC) were grown for 24 h in 100 mm dishes and transfected with 10 μg of shuttle vector, 10 μg of pGag-Pol, and 5 μg of pVSV-G using the calcium phosphate method. The transfection medium was changed after 6 h and cells were grown for 48–72 h in DMEM supplemented with 10% FCS, 2% glutamine, penicillin and streptomycin at 37 °C with 5% CO2. A total of four plates were used for each of the three independent lineages (L1–L3). Supernatants containing pseudotyped viruses were collected, filtered using a 0.45 μm filter and concentrated 40 × by centrifugation at 4,000*g* in Vivaspin20 columns. Concentrated pseudotyped viruses (1 ml) were then used to transduce 5 × 10^6^ fresh HEK 293T cells by incubation with polybrene (3.2 μg ml^−1^) in a final volume of 5 mL DMEM in a 60 mm dish for 5 h, detaching cells every hour, after which cells were transferred to a T75 flask. After 24–32 h, puromycin (0.6 μg ml^−1^) was added to select for cells in which genomic integration of the *env* or *int–vif–vpr* cassettes occurred, and selection medium was replaced every 2 days. For serial passaging, the same procedure was performed as above but transfection was carried out using the puromycin-selected cells and plasmids pGag-Pol and pVSV-G only, for a total of four (*env*) or six (*int–vif–vpr*) infection cycles. DNA was extracted from cells using DirectPCR lysis reagent following the manufacturer's recommended protocol and subjected to PCR with Phusion high-fidelity DNA polymerase and primers Env_F/Env_R and IVV_F/IVV_R, for *env* and *int–vif–vpr* fragments, respectively (Supplementary Table 1). PCR products were column purified and cloned using CloneJET PCR Cloning Kit. DNA was purified from small bacterial cultures using the Nucleospin Plasmid Kit and sequenced by the Sanger method.

### Analysis of mutations produced in the shuttle vector

The sequence of the transfected DNA construct was used to call mutations and mutation rates were calculated for each line (L1–L3) as the number of different mutations divided by sequence length and by the number of passages. The reported standard errors of the mean correspond to the among line variation (*n*=3). For mutation clustering analysis, L1–L3 were pooled, and the probability of observing a given number of mutations (*N*_obs_) in a 15 nt sliding window under the null hypothesis of a constant mutation rate was calculated as *P*=1–Bi(*N*_obs_–1|*n*, *p*=*m*/*T*), where Bi is the Binomial cumulative probability function, *n* the number of bases read for the specific window, *m* the total number of mutations in the entire sequence, and *T* the total number of bases read. To identify mutation-prone sequence motifs, for all possible di-, tri- and tetra-nucleotides the number of mutations occurring within each sequence motif at a particular base (focal base) was compared against the overall mutation rate (background rate) by means of a Fisher test, using Dunn–Sidak multiple-test correction.

### *In vitro* reverse transcription assays

A pUC18 plasmid construct containing the PSTVd sequence under the control of T3 promoter was kindly provided by Professor Ricardo Flores (IBMCP-CSIC, Spain). As a control, a PSTVd randomized sequence was synthesized *de novo* and cloned under the same promoter. Plasmid DNA was linearized with XbaI in both cases and purified using the standard sodium acetate protocol under RNAse free conditions. RNA was obtained by *in vitro* transcription of 1 μg linearized DNA using T3 RNA polymerase. After incubation (4.5 h at 37 °C), an excess of Turbo DNase and RiboLock RNase inhibitor was added to each reaction and RNA was purified with the Nucleospin RNA Clean-up XS Kit. HIV-1 RT derived from strain BH10 was kindly supplied by Professor Luis Menéndez-Arias (CBMSO-CSIC, Spain), and the reverse transcription assay was performed in 50 mM Tris-HCl (pH 8.3) containing 75 mM KCl, 3 mM MgCl_2_, 10 mM dithiothreitol, 1 U μl^−1^ RNase inhibitor, dATP, dCTP, dGTP and dTTP (250 μM each), a sequence-specific primer (0.5 μM), 100 ng template RNA and the BH10 RT (150 nM)^58^. Reactions were maintained at 42 °C for 1 h and quenched at 92 °C for 10 min. For each RNA, three independent RT reactions were performed. The cDNA was PCR-amplified using Phusion high-fidelity DNA polymerase and sequence-specific primers (500 nM each). PCR products were excised from 0.8% agarose gel and purified using the Gel DNA Recovery Kit. Cloning was performed with CloneJET PCR cloning Kit, and colony PCRs were sequenced using the Sanger method.

### Analysis of published patient sequence data

For each subtype A, B and C we selected the first 100 Blast hits using the subtype reference sequence as query and limiting the search to one sequence per patient and removing problematic and highly similar sequences. This search was performed using the HIV sequence database (www.hiv.lanl.gov). The deduced protein sequences were obtained for each reading frame and Shannon entropy at each site of the alignment (Fig. 2a) was calculated as *H*=−Σ *p*_*i*_log(*p*_*i*_), where *p*_*i*_ is the frequency of each amino acid present in the alignment, using the Entropy-One tool of the HIV sequence database. These alignments were also used to calculate the abundance of mutation-prone motifs (GG, GA, TGG and so on) in each subtype (Table 2). To infer the mutation rate from patient data using the lethal mutation method, premade curated alignments were downloaded from the HIV sequence database (see links on Table 3). Following previous work^38^,^39^, at each site of the alignment we counted the number of UGA, UAG and UAA stop codons and the number of codons that could be mutated to one of these stops by one or two different single substitutions to obtain the total number of NSMTs.

### Illumina sequencing of patient samples

Nine samples from patients were obtained from by the HIV BioBank and belong to the cohort of adults with HIV infection of the AIDS Research Network (CoRIS)^59^. CoRIS is an open, multicenter cohort of patients newly diagnosed with HIV infection in the hospital or treatment center, over 13 years of age, and naive to antiretroviral treatment. One additional sample was provided by Hospital La Fe (Valencia, Spain). Each participating patient signed an informed consent form. The programme was approved by the CoRIS Institutional Review Boards. Samples were frozen immediately after their reception, and DNA extraction was performed from 10 million PBMCs using QIAamp DNA Blood Mini Kit. Following previous work^60^, limiting-dilution nested PCR was performed to amplify clonal sequences using the Phusion high-fidelity DNA polymerase using the primers shown in Supplementary Table 2, and setting the fraction of positive PCRs to 10%. Positive reactions were mixed in equimolar pools of five, column purified and sequenced in an Illumina HiSeq2000 machine using paired-end libraries. Fastq files were cleaned, trimmed and dereplicated, and the consensus of each patient was obtained from 50,000 paired reads sampled from each library and mapped to subtype B reference sequences (HXB2, pNL4.3, K03455, AY423387, AY173951 and AY331295). In addition, a consensus sequence of each pool of five PCRs was obtained and used to calculate the number of NSMTs (that is, number of possible stop codons). Mutations were then called at each codon position, excluding those occurring only in the last 10 bases of reads. Positions with stop codons were extracted and their occurrence in each pool of five clones (1/5, 2/5, 3/5, 4/5 and 5/5) was estimated as a function of their observed frequency.

### Software

Calculations and statistics were performed with MS Excel, IBM SPSS v19, and R (www.r-project.org). Protein structure was visualized with PyMOL (www.pymol.org), and RNA structure with Qiagen CLC Main Workbench. Sanger chromatograms were edited with Staden v2.0 (staden.sourceforge.net). Sequences from the HIV database were aligned with the Muscle algorithm implemented in MEGA v5 (www.megasoftware.net). Fastq files from Illumina sequencing were cleaned and trimmed using FASTX toolkit v0.0.14 (hannonlab.cshl.edu/fastx_toolkit) and dereplicated using Prinseq-lite v0.20.3 (prinseq.sourceforge.net). Sampling, pooling and mapping of reads to obtain patient consensus sequences was done with Bowtie 2 v2.2.4 (bowtie-bio.sourceforge.net) and VICUNA. Reads were mapped to references using V-Phaser2 and MOSAIK-2.2.3 (code.google.com/p/mosaik-aligner). V-profiler was used to call mutations at each codon position. VICUNA, V-Pshaser2 and V-profiler were downloaded from www.broadinstitute.org. Gene boundary positions, as well as position of *env* V1 and V5 regions were obtained using the sequence Locator Tool from the HIV database.

## Additional information

**Accession codes:** Founder *env* and *int-vif-vpr* sequences after primer clipping were deposited in GenBank under accessions KT698943 and KT698942, respectively. A list of mutations relative to these reference sequences is provided in the, as indicated in the text.

**How to cite this article:** Geller, R. *et al*. The external domains of the HIV-1 envelope are a mutational cold spot. *Nat. Commun.* 6:8571 doi: 10.1038/ncomms9571 (2015).

## Supplementary Material

Supplementary Figures and Tables Supplementary Figures 1-2 and Supplementary Tables 1-2

Supplementary Data 1 List of *env* spontaneous mutations produced in the shuttle vector.

Supplementary Data 2 List of *int-vif-vpr* spontaneous mutations produced in the shuttle vector.

Supplementary Data 3 Sequence coordinates of *env* mutations, mutation clusters, protein domains, entropy, location of B epitopes, and previously published SHAPE data.

Supplementary Data 4 Sequence coordinates of *int-vif-vpr* mutations.

Supplementary Data 5 Nature and location of mutations in the PSTVd sequence fragment.

Supplementary Data 6 Nature and location of mutations in the randomized PSTVd sequence

Supplementary Data 7 Number and sequence coordinates of stop codons in datasets 1 and 2 from Table 3.

Supplementary Data 8 Number of stop codons and their location in sequences from viral DNA isolated from 10 patients.

## Figures and Tables

**Table 1:** HIV-1 rate and spectrum of point mutations*.

Replicate	*env* (2,598 nt)			*int–vif–vpr* (1,566 nt)		
	1	2	3	1	2	3
G→A	20	10	18	18	15	19
Other transitions	10	3	3	10	4	9
Transversions	8	4	17	6	6	8
Insertions	3	4	2	0	3	2
Deletions	0	0	2	1	1	0
Total mutations	41	21	42	35	29	38
Bases sequenced	246,281	222,208	248,935	75,168	75,168	76,734
Infection cycles	4	4	4	6	6	6
Mutation rate (m/n/c)	4.2 × 10^−5^	2.4 × 10^−5^	4.2 × 10^−5^	7.8 × 10^−5^	6.4 × 10^−5^	8.3 × 10^−5^

^*^A list of all mutations is provided in Supplementary Data 1 (*env*) and Supplementary Data 2 (*int–vif–vpr*).

**Table 2:** Mutation-prone sequence motifs in HIV-1 *env*.

Motif*	Fraction mutated†	Odds ratio‡	Likely source
GG	17/166	2.9***	A3G
TGG	9/64	3.9**	
GGA	12/67	5.0***	
TGGA	8/26	8.6***	
TTGG	6/13	12.8***	
			
GA	23/202	3.2***	A3D/F/H
GAA	17/73	6.5***	
TGA	10/38	7.3***	
GGAA	5/23	6.0**	
ATGA	4/10	11.1***	
GTGA	5/9	12.3***	
TGAA	9/15	16.7***	
			
TG	26/183	4.0***	A3/RT
TTG	9/47	5.3***	
ATG	10/50	5.6***	
TTCT	4/11	10.11**	

^*^The mutated base is underlined.

^†^Mutated/total motifs in *env*.

^‡^Fold mutation rate increase relative to the background rate. Asterisks indicate statistical significance, based on Fisher's exact test using Dunn–Sidak multiple test correction (*P<0.05, **P<0.01, ***P<0.001 after correction).

**Table 3:** Intrapatient stop codon frequencies stemming from likely A3 and RT mutations.

	Plasma dataset 1 (2,908 sequences)*		Plasma dataset 2 (1,573 sequences)†		Proviral dataset (500 sequences)‡	
	**gp120 OA**	**Rest of** ***env***	**gp120 OA**	**Rest of** ***env***	**gp120 OA**	**Rest of** ***env***
Premature stop codons	12	41	5	24	162	1,322
Available targets (NSMTs)	379,586	753,733	200,679	406,084	68,550	124,500
Stop codon frequency	3.2 × 10^−5^	5.4 × 10^−5^	2.5 × 10^−5^	5.9 × 10^−5^	2.4 × 10^−3^	1.1 × 10^−2^

^*^Subtype B sequences (www.hiv.lanl.gov/content/sequence/HIV/USER_ALIGNMENTS/keele.html), see Supplementary Data 7 for details.

^†^Subtype C sequences (www.hiv.lanl.gov/content/sequence/HIV/SI_alignments/set5.html), see Supplementary Data 7 for details.

^‡^Data from this study, see Supplementary Data 8 for details.
