# Supplementary material for: The external domains of the HIV-1 envelope are a mutational cold spot
Source: Nat Commun. 2015 Oct 9;6:8571. doi: 10.1038/ncomms9571 (PMC4687473; doi:10.1038/ncomms9571)
Supplement: Supplementary Figures and Tables — Supplementary Figures 1-2 and Supplementary Tables 1-2 [file ncomms9571-s1.pdf]

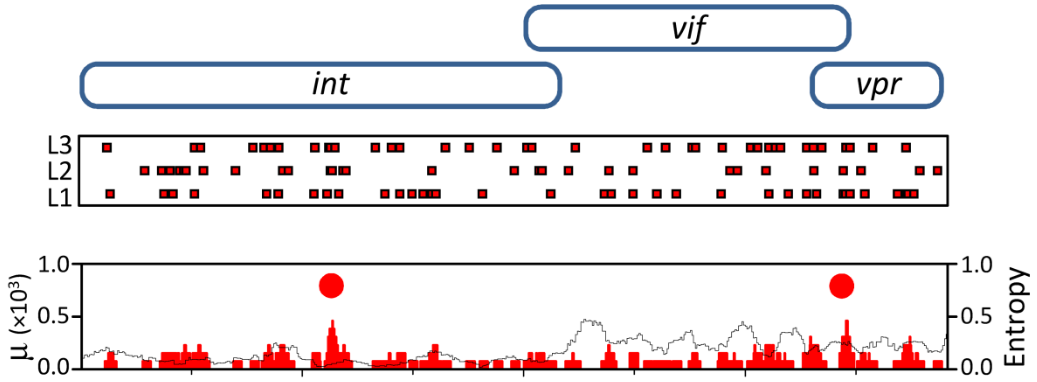


**Supplementary Figure 1. Location of spontaneous mutations across the *int-vif-vpr* region.** On top is shown the genetic map of the region. Nucleotide substitutions found for each of the lines L1-L3 after four infection cycles are show as red squares (middle). Bottom: mutation rate (μ) averaged over 15-base sliding window (red bars). Significant mutation clusters are indicated with red circles.

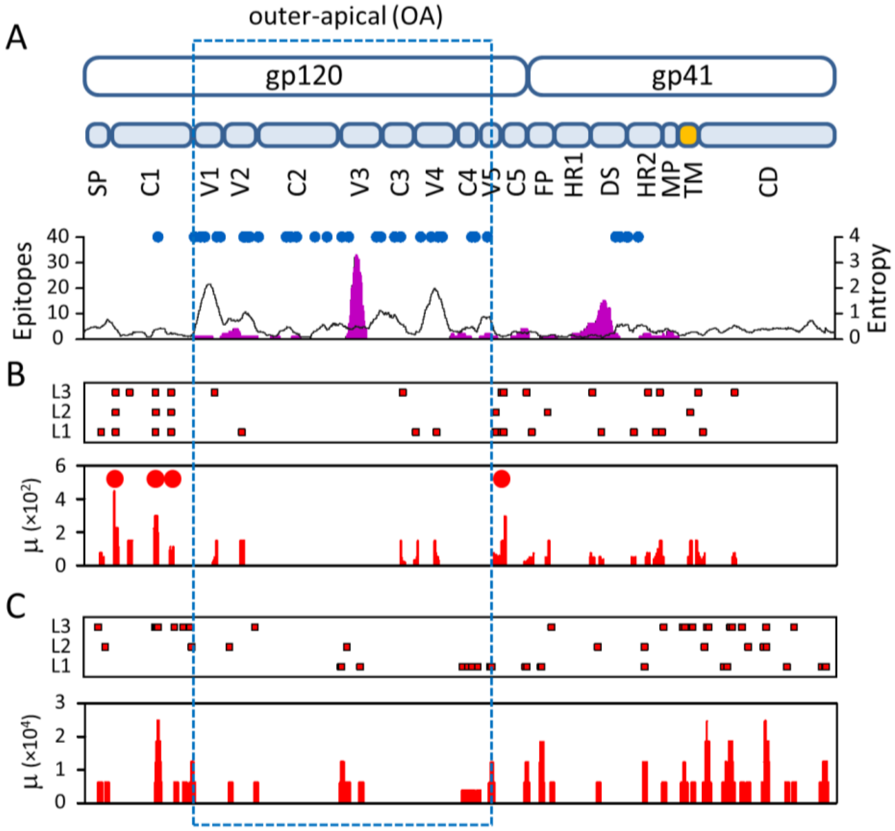


**Supplementary Figure 2. Location of A3-like and RT mutations across *env*.** The *env* genetic map is shown on top (**A**). Abbreviations are defined in Figure 2 legend. The 1 kb region encoding the extensively glycosylated outer-apical domains of gp120 is boxed, and glycosylation sites (blue dots), number of B-cell epitopes (pink histogram), and protein sequence variability calculated as the Shannon entropy averaged over a 15-residue sliding window (black skyline) are indicated. **B.** A3-like (G→A substitutions in <u>G</u>G or <u>G</u>A motifs) found in each of the lines L1-L3 are shown with red squares. Red bars indicate the mutation rate ($\mu$) averaged over 15-base sliding window. Notice that the per-target mutation rate is considerably higher than for non-A3 mutations. A3 targets that were mutated more than once in different lines are shown with red circles. **C.** Substitutions attributed to the HIV-1 RT mutations (all except A3-like mutations) in each of the lines L1-L3 are shown with red squares. Red bars indicate the mutation rate ($\mu$) averaged over 15-base sliding window.

**Supplementary Table 1.** Shuttle vector primers used for cloning and PCR.

| Name | Orientation | Sequence (5'-3') |
|---|---|---|
| Env_F_Mlu | Forward | ACCCGACACGCGTTGGCAATGAGAGTGATGGG |
| Env_R_Xho | Reverse | GACTAGCTCGAGTTTTGACCACTTGCCACCC |
| IVV_F_Mlu | Forward | ACCCGACACGCGTTTTTTAGATGGGATAGATAAG |
| IVV_R_Xho | Reverse | GACTAGCTCGAGGCTATGTTGGCACCCAATTC |
| Env_F | Forward | GGCAATGAGAGTGATGGGGA |
| Env_R | Reverse | TTTTTGACCACTTGCCACCCA |
| IVV_F | Forward | TTTTTAGATGGGATAGATAAGGCTC |
| IVV_R | Reverse | GCTATGTTGGCACCCAATTC |

**Supplementary Table 2.** Primers used for nested limiting-dilution PCR of patient samples.

| Patients | Name | Orientation | Sequence (5'-3') | HXB2 location |
|---|---|---|---|---|
| All except | 294TU | Forward | ATGGCTTAGGGCAACATATCTATG | 5677-5700 |
| R14-R115 | 163U5D | Reverse | CTGAGGGATCTCTAGTTACCAGAG | 9664-9687 |
| | 551VU | Forward | GGAAGCCATAATAAGAATTCTGCAACAACTGC | 5729-5760 |
| | 555U5 | Reverse | CCAGAGTCACACAACCGCGGGGCACACACTACT | 9637-9669 |
| R14 | 3'HIV-O-S | Forward | CAAAATTTTCGGGTTTATTACAGGGACA | 4890-4917 |
| | 163U5D | Reverse | CTGAGGGATCTCTAGTTACCAGAG | 9664-9687 |
| | 294TU | Forward | ATGGCTTAGGGCAACATATCTATG | 5677-5700 |
| | 555 | Reverse | CCAGAGTCACACAACCGCGGGGCACACACTACT | 9637-9669 |
| R15 | 3'HIV-O-S 3 | Forward | TACAGTGCAGGGGAAAGAATAATAGACATAATA | 4809-4834 |
| | 555U5 | Reverse | CCAGAGTCACACAACCGCGGGGCACACACTACT | 9637-9669 |
| | 551 | Forward | GGAAGCCATAATAAGAATTCTGCAACAACTGC | 5729-5760 |
| | 2R3.B6R | Reverse | TGAAGCACTCAAGGCAAGCTTTATTGAGGC | 9607-9636 |
